# Formative pluripotency: the executive phase in a developmental continuum

**DOI:** 10.1242/dev.142679

**Published:** 2017-02-01

**Authors:** Austin Smith

**Affiliations:** Wellcome Trust-Medical Research Council Stem Cell Institute and Department of Biochemistry, University of Cambridge, Tennis Court Road, Cambridge CB2 1QR, UK

**Keywords:** Embryonic stem cells, Pluripotency, Epiblast, Lineage specification, Developmental potential

## Abstract

The regulative capability of single cells to give rise to all primary embryonic lineages is termed pluripotency. Observations of fluctuating gene expression and phenotypic heterogeneity *in vitro* have fostered a conception of pluripotency as an intrinsically metastable and precarious state. However, in the embryo and in defined culture environments the properties of pluripotent cells change in an orderly sequence. Two phases of pluripotency, called naïve and primed, have previously been described. In this Hypothesis article, a third phase, called formative pluripotency, is proposed to exist as part of a developmental continuum between the naïve and primed phases. The formative phase is hypothesised to be enabling for the execution of pluripotency, entailing remodelling of transcriptional, epigenetic, signalling and metabolic networks to constitute multi-lineage competence and responsiveness to specification cues.

## Abstract (precis)

**Summary:** This Hypothesis article poses that a third state of pluripotency, called formative pluripotency, exists between the naïve and primed states, and is enabling for the execution of pluripotency.

## Introduction

**Fig. 1.**
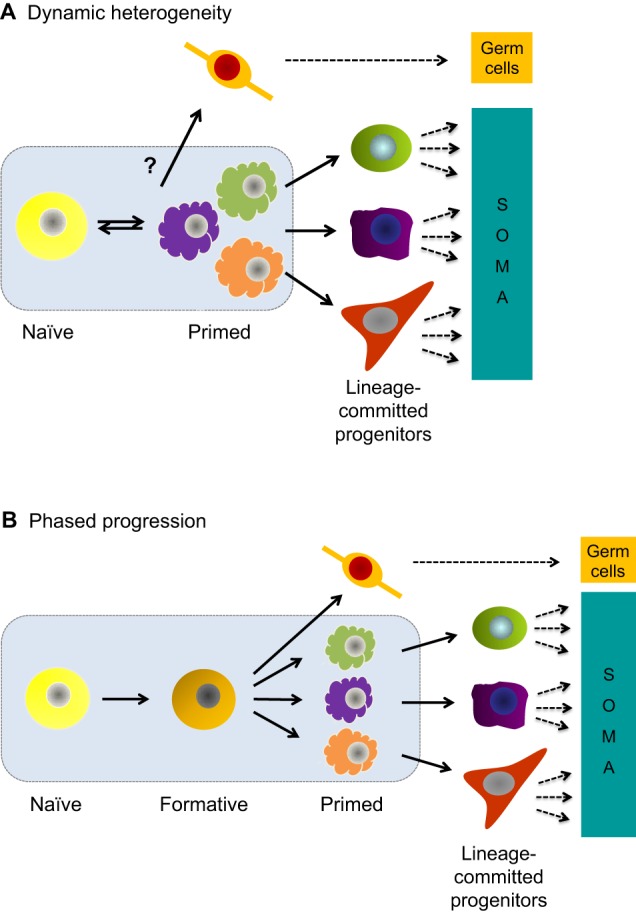
**Dynamic heterogeneity and phased progression models of pluripotency.** (A,B) In the dynamic heterogeneity model of pluripotency (A), naïve and metastable primed cell states co-exist and are interconvertible. Fluctuation between states creates windows of opportunity for commitment. Germline segregation is not well-delineated within this framework. In the phased progression model of pluripotency (B), cells transit sequentially through naïve to formative to primed forms of pluripotency en route to lineage commitment. In the embryo, this process is an orderly continuum. *Ex vivo*, however, ESCs cultured in serum may comprise all phases simultaneously and the unidirectional developmental order may even be reversed, creating a situation similar to the dynamic heterogeneity model. In both models, culture of mouse ESCs in 2iLIF ground-state conditions constrains pluripotency within the naïve phase. Dashed lines indicate multi-step differentiation, blue shading represents the Oct4-positive pluripotent populations.

Pluripotency may be defined as an intrinsic and flexible cellular potential to generate all cell lineages of the mature organism. Recently, pluripotency has been described in two forms: naïve and primed (Hackett and Surani, 2014; Nichols and Smith, 2009). These terms refer to pre- and post-implantation populations in the embryo and their associated *in vitro* stem cell states. Naïve and primed pluripotent cells are often presented as directly inter-convertible (Fig. 1A), based on observations *in vitro* of heterogeneity and reprogramming. However, the two-stage model is an over-simplification that omits a pivotal developmental transformation. Pluripotency may be viewed more accurately as a developmental progression through consecutive phases (Fig. 1B). In this article, the hypothesis presented is that between naïve and primed pluripotency, a formative interval is mandatory to acquire competence for multi-lineage induction. There are two corollaries to this hypothesis: first, that naïve pluripotent cells are unprepared to execute lineage decisions and must necessarily undergo a process of maturation; and, second, that primed cells have initiated a response to inductive cues and are already partially specified and fate-biased. Characterisation of the formative phase is posited to be crucial for understanding the conditions for, and mechanisms of, multi-lineage decision-making.

## Naïve and primed pluripotency: the epiblast, ESCs and EpiSCs

**Fig. 2.**
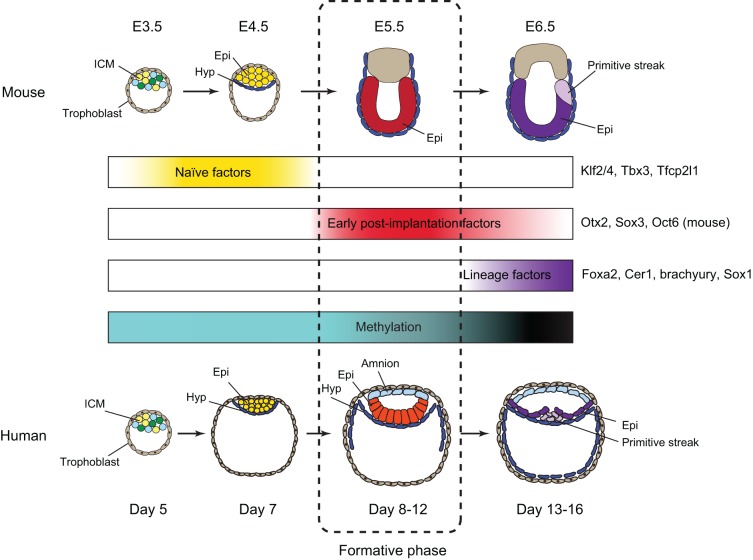
**Developmental progression of pluripotency in mouse and human embryos.** Pluripotent cells begin to emerge in the ICM and segregate to constitute the naïve epiblast. The multi-coloured cells of the ICM indicate mosaic specification of epiblast and hypoblast. After implantation in both mouse (E5) and human (day 8) embryos the epiblast expands as a pseudoepithelial layer overlying the hypoblast (also called the extra-embryonic endoderm), forming a cup-shaped cylinder in mice and a disc in humans. During this period, epiblast cells may remain unpatterned and without molecular specification. Subsequently, epiblast cells become fixed in a columnar epithelium, display regionalised expression of specification factors in response to extra-embryonic signalling centres, and initiate gastrulation. This sequence of events is reflected in transcriptional and epigenetic changes. The distinction between naïve pluripotency and the hypothesised formative phase appears to be acute, whereas the subsequent transition to primed pluripotency is more gradual. Formative and primed phases may be present together at the early stages of gastrulation, particularly in humans. Epi, epiblast; Hyp, hypoblast.

In the embryos of eutherian mammals, pluripotency emerges within the inner cell mass (ICM) of the blastocyst and persists until somitogenesis (Osorno et al., 2012). Over this period, lasting 4-5 days in mouse and approximately two weeks in human embryos, cells in the pluripotent tissue, the epiblast, alter their cellular properties and undergo global transformations in transcriptomic and epigenomic features (Fig. 2) plus changes in signalling and metabolism. An initial group of around ten apparently homogeneous epiblast cells in the mouse ICM proliferates after implantation and develops by the onset of gastrulation into several hundred cells. Gastrula-stage epiblast cells are individually fated and molecularly specified according to their location, but are not yet committed (Lawson et al., 1991; Peng et al., 2016; Tam and Zhou, 1996). The pluripotent epiblast continues to expand during early to mid-gastrulation but by the onset of somitogenesis all cells have restricted potency (Osorno et al., 2012). The highly regulative character of the epiblast is illustrated by the natural occurrence in many mammals of pre-implantation diapause – a facultative delay before uterine implantation (Renfree and Shaw, 2000) – and by classical embryological perturbations and transplantations. For example, the epiblast can rapidly adjust to dramatic increases or reductions in cell number (Buehr and McLaren, 1974; Gardner and Beddington, 1988; Lewis and Rossant, 1982; Rands, 1986a,b; Snow and Tam, 1979), and cells in the late epiblast that are fated and express different combinations of lineage-affiliated transcription factors can be re-specified by heterotopic grafting (Beddington, 1983; Tam and Zhou, 1996). Such remarkable regulative capacity implies a highly malleable gene regulatory circuitry. This flexibility may incidentally provide the facility for *ex vivo* propagation of stem cells from a dynamic tissue that, in the strictest sense, does not self-renew.

The defining attribute of mouse embryonic stem cells (ESCs) is the ability to colonise the blastocyst and contribute extensively to all lineages of resulting chimaeric animals, including production of functional gametes (Bradley et al., 1984). Mouse ESCs self-renew rapidly and continuously *in vitro*. A key signal for sustaining their self-renewal is the cytokine leukaemia inhibitory factor (LIF) (Smith et al., 1988; Williams et al., 1988), which activates the transcription factor Stat3 (Niwa et al., 1998). Unusually, ESCs do not utilise mitogen-activated protein kinase (Erk1/2; also known as Mapk3/Mapk1) signalling for cell cycle progression. Selective blockade of this pathway suppresses differentiation (Burdon et al., 1999) without impeding propagation (Ying et al., 2008). Additional partial suppression of glycogen synthase kinase 3 (GSK3) further blocks differentiation (Wray et al., 2011). Together, suppression of phospho-Erk and GSK3 activity is sufficient for ESC maintenance, even without LIF in some genetic backgrounds (Wray et al., 2010; Ying et al., 2008). Use of this two inhibitor (2i) system plus LIF captures naïve pluripotency as a discrete *in vitro* state, sometimes called the pluripotent ground state (Marks et al., 2012; Ying et al., 2008). Importantly, this system has made ESC derivation highly consistent and applicable to different strains of mice (Kiyonari et al., 2010; Nichols et al., 2009), and also to rats (Buehr et al., 2008; Li et al., 2008). Thus, ESC production appears to reflect a generic property of the pre-implantation epiblast in these species. Indeed, ESCs show strong transcriptome-wide similarity to the newly formed epiblast at mouse embryonic day (E) 3.75-4.5 (Boroviak et al., 2014, 2015).

The ability to derive mouse ESCs declines precipitately in the peri-implantation period (Boroviak et al., 2014; Brook and Gardner, 1997). This is in spite of the fact that the epiblast expands continuously after implantation and will readily give rise to teratocarcinomas and derivative pluripotent embryonal carcinoma cells (Solter et al., 1970; Stevens, 1970). Explants of post-implantation epiblast can give rise to stem cells if cultured in conditions different to those for ESCs, however. Use of fibroblast growth factor (FGF) and activin instead of LIF enabled establishment of a pluripotent cell type named post-implantation epiblast-derived stem cells (EpiSCs) (Brons et al., 2007; Tesar et al., 2007). EpiSCs can be derived from the epiblast between E5.5 and E8.0 (Osorno et al., 2012). They are heterogeneous but converge on a global transcriptome with features of late gastrula-stage epiblast (Kojima et al., 2014; Tsakiridis et al., 2014). EpiSCs do not integrate well into the ICM and therefore fail to produce substantial chimaerism after morula or blastocyst injection. Importantly, however, when grafted into post-implantation epiblast in whole embryo culture, EpiSCs show evidence of incorporation into developing germ layers (Huang et al., 2012; Kojima et al., 2014; Tsakiridis et al., 2014). Furthermore, transgenic expression of Bcl2 enables EpiSCs to survive after injection into the pre-implantation embryos and subsequently to colonise the egg cylinder (Masaki et al., 2016). This and other genetic manipulations enable EpiSC contribution to somatic tissues in chimaeras, although apparently not to the germ line (Ohtsuka et al., 2012).

Thus, it has so far proven possible to pause developmental progression of the rodent epiblast at initial and late phases of pluripotency and to establish two different stem cell states *in vitro*. The terminology ‘naïve’ and ‘primed’ was introduced to underscore the recognition that pluripotency is not a unitary state (Nichols and Smith, 2009).

## Dissolution of naïve pluripotency precedes lineage priming

Cells exiting the naïve ESC ground state *in vitro* show early morphological changes, involving both cell movement and flattening within 20 h in adherent culture (Kalkan et al., 2016 preprint) and the formation of rosette structures in 3D culture (Bedzhov and Zernicka-Goetz, 2014). These events are potentially significant for biomechanical responsiveness and extracellular matrix signalling. *In utero*, the epiblast undergoes a morphogenetic transformation shortly after implantation. An amorphous cell cluster converts into a cup-shaped monolayer residing on a basement membrane. Clonal analysis indicates a high degree of cell mixing at E5.0-6.0 (Gardner and Cockroft, 1998), which suggests that epithelialisation is incomplete and cells can detach from the basal lamina during mitosis and leave the epithelial layer temporarily before re-integrating. Continuous dispersion and re-association might be important to avoid premature specification when localised patterning centres begin to form in the extra-embryonic tissues (Beddington and Robertson, 1999). Mixing might also facilitate elimination of unfit cells through cell competition (Sancho et al., 2013). By contrast, a day later at the pre-streak stage cell fate can be mapped reliably from location, meaning that epithelial integrity is consolidated and cell positions are fixed (Lawson et al., 1991; Tam and Zhou, 1996). By that time, the egg cylinder is pseudo-stratified with overt apicobasal polarity. These cell biological changes might be reflected in discriminating features such as tight junction density or Rho kinase activity.

Mouse pre-implantation epiblast and ESCs are characterised by co-expression, along with Oct4 (Pou5f1) and Sox2, of a suite of transcription factors including Klf4, Tfcp2l1, Esrrb, Klf2, Tbx3, Prdm14 and Nanog that are absent from the immediate post-implantation epiblast (Boroviak et al., 2015). Collectively, these transcription factors constitute a flexible control circuitry that sustains ESC self-renewal (Chen et al., 2008; Dunn et al., 2014; Martello and Smith, 2014; Niwa et al., 2009). Dissolution of this circuitry is evident from the onset of ESC differentiation (Kalkan et al., 2016 preprint; Kalkan and Smith, 2014). Efficient clearance of the naïve transcription factors extinguishes ESC self-renewal capacity and enforces loss of ESC identity. Genetic screens have implicated multiple pathways in the clearance process, acting transcriptionally and post-transcriptionally (Betschinger et al., 2013; Leeb et al., 2014). As the naïve factors disappear, a reciprocal gain of expression is apparent for Otx2 and Oct6 (Pou3f1) (Kalkan et al., 2016 preprint). These events at the onset of ESC differentiation recapitulate expression dynamics in the late blastocyst whereby Nanog is extinguished before implantation and Otx2 and Oct6 are upregulated throughout the epiblast (Acampora et al., 2016; Chambers et al., 2003). Other factors for which expression is upregulated early *in vivo* and *in vitro* include Sox3, Sall2, the growth factor Fgf5, and the *de novo* methyltransferases Dnmt3a and Dnmt3b (Boroviak et al., 2015; Kalkan et al., 2016 preprint). Upregulation of Dnmts is accompanied by a substantial genome-wide increase in CpG methylation, *in vivo* and *in vitro*, to a level intermediate between the pre-implantation ICM or ESCs and the E6.5 epiblast (Auclair et al., 2014; Kalkan et al., 2016 preprint; Seisenberger et al., 2012).

Significantly, the expression of factors considered to denote lineage specification, such as brachyury, Foxa2 or Sox1, is not evident in cells that have newly and irreversibly exited the ESC ground state, but only becomes appreciable at later time points (Kalkan et al., 2016 preprint; Turner et al., 2014; Zhang et al., 2010; Mulas et al., 2016 preprint). This temporal sequence of gene expression also mirrors events in the embryo. Non-neural lineage specification markers only begin to emerge at the pre-streak stage (E6.0-6.25) in local regions of the epiblast (Russ et al., 2000). Around this time, Nanog is re-expressed in the posterior epiblast (Hart et al., 2004), and Otx2, Oct6 and Sox2 subsequently become restricted to the prospective neuroectoderm in the anterior epiblast where Sox1 is upregulated. Thus, during both ESC entry into differentiation *in vitro* and epiblast progression *in utero*, there is a substantial interval of 24 h or longer between the loss of naïve pluripotency and the overt manifestation of lineage priming. During this interval, the pluripotent population might be relatively homogenous in identity, although this has yet to be examined in detail.

## Competence for lineage allocation: the requirement for a formative phase

The observed temporal separation between exit from naïve pluripotency and fate allocation might be essential for the realisation of multi-lineage potential. The central hypothesis of this article is that a formative period is obligatory for remodelling of the genomic and epigenomic blank canvas of the naïve epiblast to constitute a substrate for lineage specification. Reconfiguration entails not only handover to a distinct gene regulatory network but also increased engagement with epigenetic regulators, rewiring of signalling pathways, and a switch to predominantly glycolytic metabolism (Kalkan et al., 2016 preprint). In addition, partial epithelialisation and increased interaction with the extracellular matrix are expected to modulate signalling. These events together are envisaged to prepare a template for responsiveness to inductive stimuli and execution of lineage decisions.

A paradigm for the concept of remodelling pluripotency to prepare for fate allocation is provided by specification of the germ line. The ability to give rise to germ cells in chimaeras is a hallmark property of mouse ESCs (Bradley et al., 1984). However, ESCs themselves are refractory both to transcription factor determinants and to inductive growth factor cues for germ cell fate (Hayashi et al., 2011; Magnúsdóttir et al., 2013). To become responsive they must lose ESC identity and convert over 24-48 h to a population that has been called epiblast-like cells (EpiLCs) (Hayashi et al., 2011). Transcriptome analyses indicate that EpiLCs are distinct from EpiSCs and resemble the pre-gastrulation E5.5-E6.0 epiblast. Epiblasts at this time are highly responsive to primordial germ cell induction (Ohinata et al., 2009), unlike earlier populations. Importantly, EpiLC cultures can also respond to germ cell specification stimuli. Competence for germline induction is thus not a constitutive feature of pluripotency but is a property acquired during developmental progression. It should also be noted that germline competence is lost in mouse EpiSCs and the late epiblast (Hayashi et al., 2011). Importantly, however, competence appears to be a feature of many, if not all, epiblast cells at E5.5-6.0 (Ohinata et al., 2009), even though only a handful will become specified in normal development. A molecular correlate of germline competence is enhancer remodelling and altered transcription factor occupancy at genes that will be re-expressed in primordial germ cells (Murakami et al., 2016).

Germ cell competence is acquired in the interval between naïve and primed pluripotency. The hypothesis of a formative phase postulates that during this interval competence is also installed for somatic lineage specification. A shared requirement for the formative phase would be consistent with the close association between germ cell and somatic specification from pluripotent founders in divergent mammals (Irie et al., 2015; Johnson and Alberio, 2015) and the key requirement for Blimp1 (Prdm1) to repress somatic fates in order to enable germline specification (Hayashi et al., 2007). The formative remodelling of pluripotency is thus proposed to generate a group of equivalent cells that are uniformly equipped to respond to patterning and lineage specification cues. The neural default model argues that neuroectodermal competence is the fate pluripotent cells will adopt if not instructed otherwise (Muñoz-Sanjuán and Brivanlou, 2002). The formative hypothesis can be compatible with this idea and indeed it is noteworthy that transcription factors that might be key to formative pluripotency, such as Otx2, Oct6 and Sox3, subsequently become restricted to the anterior primed epiblast and thence to the neuroectoderm.

## Criteria for evaluating formative pluripotency

The formative epiblast is proposed as the launching pad for multi-lineage differentiation. Several properties of formative pluripotency are expected, relative to attributes of the naïve and primed phases (see Table 1). Predictions can be made for the outcomes of forthcoming single-cell transcriptomic and epigenomic characterisation of the early post-implantation mouse epiblast and of ESC differentiation trajectories.

**Table 1.**
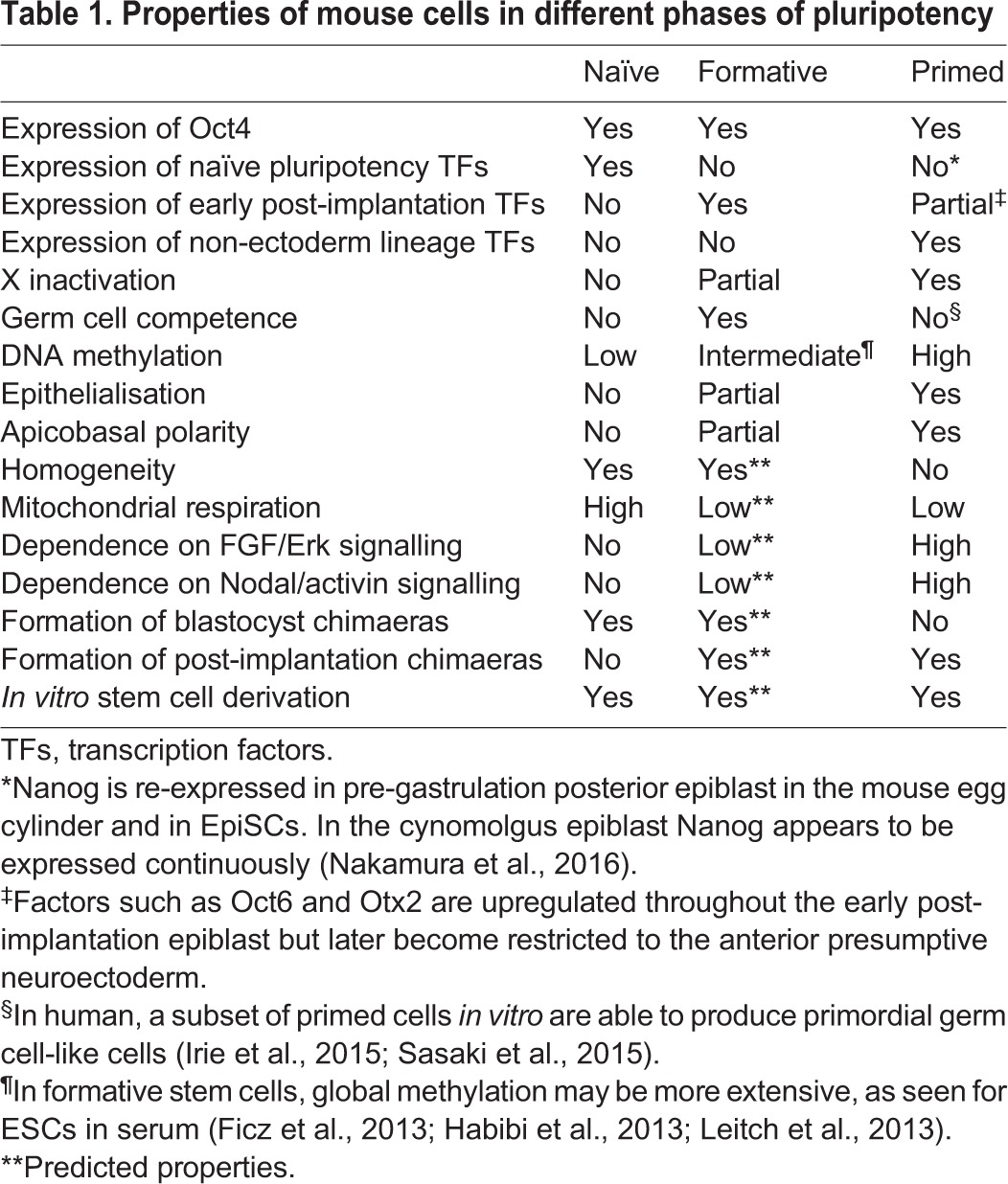
**Properties of mouse cells in different phases of pluripotency**

In terms of global gene expression, formative cells should occupy a transcriptional state space intermediate between naïve and primed populations. The repertoire of expressed genes is expected to be similar in all formative cells, although intrinsic noise in gene expression levels might be greater than in naïve cells due to a more permissive chromatin context. Neither naïve factors nor those that specify a given lineage should be substantially expressed. Regionalised gene expression should be minimal and/or inconsequential in the embryo due to cell dispersion. The formative gene regulatory network is expected to have the general pluripotency factors Oct4 and Sox2 at its core. In the mouse, Otx2 is likely to be a key factor because it is rapidly upregulated *in vivo* and *in vitro* (Acampora et al., 2013, 2016; Kalkan et al., 2016 preprint) and co-occupies newly commissioned enhancers with Oct4 (Buecker et al., 2014; Yang et al., 2014). Based on their co-incident upregulation in mouse, significant roles may also be anticipated for Oct6 and Sox3, but other key factors remain to be defined. The specific transcriptional regulators are likely to differ between mice and primates, however (Nakamura et al., 2016).

It is anticipated that the transition into formative pluripotency should entail a profound reconstruction of the chromatin landscape (Zylicz et al., 2015). Global increases in both DNA methylation and polycomb-mediated deposition of H3K27me3 compared with naïve cells (Auclair et al., 2014; Buecker et al., 2014; Kalkan et al., 2016 preprint) are expected to be accompanied by an increased number of bivalent promoters – that is, promoters that bear histone modifications associated with both silencing and activation. Notably, these events are all apparent when mouse ESCs are transferred from 2iLIF to serum (Ficz et al., 2013; Habibi et al., 2013; Leitch et al., 2013; Marks et al., 2012). Enhancer commissioning should be widespread along with selective decommissioning (Acampora et al., 2016; Buecker et al., 2014; Factor et al., 2014; Murakami et al., 2016; Yang et al., 2014). Engagement of chromatin remodellers and epigenetic regulators will equip formative cells for lineage priming. It is noteworthy that mutation of several chromatin remodellers impairs ESC lineage specification *in vitro* and compromises, or completely disables, gastrulation *in vivo* (Hu and Wade, 2012).

The generation of formative pluripotency is expected to depend on specific signalling inputs. Candidate pathways from ligand expression in the embryo are mitogen-activated protein kinase Erk1/2 signalling downstream of FGFs and integrins, and Smad2/3 activity downstream of Nodal (Robertson, 2014). However, these signals also drive lineage specification and at high levels can sustain primed EpiSCs and conventional human pluripotent stem cells (PSCs) (Vallier et al., 2005). The formative phase might therefore be characterised by low threshold activation of these pathways combined with absence or inhibition of other inductive inputs, notably Wnt (Tsakiridis et al., 2014).

The essence of the formative pluripotency concept is that it is necessary for a cell to transit through this remodelling phase in order to prepare for the proper segregation of all definitive embryonic lineages. Therefore, during *in vitro* differentiation a discrete interval should normally be present between loss of naïve characteristics and the emergence of lineage-specific features. Such a window is apparent at the onset of multi-lineage differentiation of mouse ESCs following withdrawal from 2i in adherent culture (Kalkan et al., 2016 preprint; Mulas et al., 2016 preprint) and a similar formative period might be evident during the early development of embryoid bodies (Rathjen et al., 2002; Shen and Leder, 1992) and gastruloids (van den Brink et al., 2014). Transcription factor-enforced differentiation might conceivably over-ride requirements for a formative phase. In such circumstances, however, the stability and fidelity of mature phenotypes might be compromised due to incomplete epigenetic programming. It should also be noted that extra-embryonic endoderm and trophectoderm do not originate from the epiblast during embryo development, and although some reports suggest they may be generated from ESCs *in vitro* (Morgani et al., 2013), this would not proceed via the formative phase.

During the formative interval, pluripotent cells have lost naïve identity, and are in the process of acquiring full capability in germline and somatic fate options. Indeed, formative phase cells are expected to become sensitised to lineage-inductive cues. Therefore, they should respond more rapidly and uniformly than naïve cells to inductive cues. However, in the formative phase prospective fates may readily be re-directed by alternative stimuli. Only as specification proceeds and cells become primed for individual lineages through expression of key transcription factors will heterogeneity and lineage bias emerge and re-direction to alternative fates become less readily achieved.

## Reconciling phased progression with dynamic heterogeneity

Developmental progression through pluripotency may be paused or even reverted by extrinsic conditions. This may occur during regulative compensation *in utero* (Gardner and Beddington, 1988; Snow and Tam, 1979) and during establishment of PSCs *in vitro* (Bao et al., 2009; Boroviak et al., 2014). Culture environments may also corrupt developmental trajectories, however. Mouse ESCs cultured traditionally in the presence of foetal calf serum display heterogeneous expression of multiple genes, including both transcription factors, such as Nanog, Esrrb and Klf4, which are functionally relevant to naïve pluripotency, as well as factors such as brachyury and Foxa2, which are associated with lineage specification (Marks et al., 2012; Torres-Padilla and Chambers, 2014). Furthermore, it has been demonstrated that loss of expression of certain naïve pluripotency markers can be reversible in these culture conditions (Chambers et al., 2007; Hayashi et al., 2008; Toyooka et al., 2008). These observations of dynamic heterogeneity and the co-existence of naïve and primed features in ESC cultures in serum have been interpreted as reflective of inherent metastability in pluripotent cells (Hayashi et al., 2008; Silva and Smith, 2008). It has been suggested that PSCs exist in a condition of ‘precarious balance’ (Loh and Lim, 2011) that simultaneously allows self-renewal and poising for differentiation. This metastable condition is attributed to fluctuating expression of transcription factors that may be mutually antagonistic (Herberg et al., 2016; Kalmar et al., 2009). However, the dynamic heterogeneity of ESCs in serum does not match well with trajectories observed in the embryo or in defined conditions *in vitro*, as discussed above. Furthermore, the measured periods between loss and re-expression of naïve factors extend over several cell cycles (Filipczyk et al., 2015), which is not consistent with embryonic timescales. Moreover, many cells that lose naïve factor expression do not revert (Chambers et al., 2007; Filipczyk et al., 2015; Nakai-Futatsugi and Niwa, 2016). Finally, it should also be noted that EpiSCs, which may be considered fully converted to the primed phase, do not revert to naïve ESCs when cultured in serum and LIF (Guo et al., 2009). Interestingly, however, some EpiSC lines derived on feeders show low frequency conversion to an ESC phenotype when transferred to 2iLIF (Bernemann et al., 2011; Greber et al., 2010; Han et al., 2010). One possibility is that those heterogeneous cultures could contain within them cells that are in, or close to, the formative phase and can be reset in appropriate culture conditions.

Population-based analyses of ESC cultures in serum have generally been considered in the framework of metastability and interconvertibility between naïve and primed conditions (Fig. 1A). However, the mixed and dynamic composition of these cultures could blur boundaries and obscure the proper developmental phasing (Fig. 1B). Single-cell imaging and ‘omic approaches can be applied to deconvolute population structure (Filipczyk et al., 2015; Kolodziejczyk et al., 2015; Singer et al., 2014) and inform on the presence or absence of discrete naïve, formative and primed subpopulations. Nonetheless, perturbation by serum factors might generate degrees of metastability that are not found *in vivo* and confound developmental order. In my opinion, it is time to move beyond serum for the characterisation of pluripotency and unambiguous delineation of the lineage decision-making process.

## Relevance to human embryo development and human PSCs

In 1998, Thomson and colleagues derived PSCs from human embryos (Thomson et al., 1998). These cells were considered to be human counterparts of mouse embryonic stem cells because of their blastocyst origin. However, from the outset differences were apparent. The current consensus is that human ICM cells develop to a post-implantation embryonic disc stage in primary explants (O'Leary et al., 2012) and derivative cell lines are more similar to primed EpiSCs than to naïve ESCs (Davidson et al., 2015; De Los Angeles et al., 2012; Rossant, 2015). For example, transcription factors KLF4 and TFCP2L1 function in mouse ESC self-renewal (Dunn et al., 2014; Martello et al., 2013; Niwa et al., 2009; Ye et al., 2013) and both are present in human ICM (Takashima et al., 2014) but extinguished during explant culture and establishment of conventional human PSCs (O'Leary et al., 2012). The hypomethylated ICM genome is also rapidly methylated during ICM explant culture (Smith et al., 2014).

Human PSCs, whether embryo-derived or generated by molecular reprogramming (Takahashi et al., 2007; Yu et al., 2007), are conventionally maintained in similar culture conditions as EpiSCs, and do not tolerate 2iLIF. Recently, however, progress has been made in generating PSCs that do expand in variants of 2iLIF media and exhibit a range of features consistent with a more naïve identity (Davidson et al., 2015; Huang et al., 2014; Theunissen et al., 2016). This has been achieved both by resetting established human primed pluripotent cells (Takashima et al., 2014; Theunissen et al., 2014), and by direct derivation from dissociated human ICM cells (Guo et al., 2016). Although further investigation is required to determine the optimal culture conditions and the precise relationship between human naïve cells *in vivo* and *in vitro*, the present findings lend support to the premise of conserved principles of pluripotency progression in eutherian mammals, although specific functional attributes might vary (Boroviak and Nichols, 2017).

It follows that a phase of formative pluripotency should be identifiable in primates. Unlike rodents, but in common with other mammals, primate embryos do not develop via an egg cylinder. Instead, the epiblast and hypoblast form a bilaminar disc (Boroviak and Nichols, 2017; Rossant, 2015). In primate embryos, this structure persists for several days prior to gastrulation. It has been poorly characterised due to limitations in accessing early post-implantation material. However, a recent landmark study has now provided the first transcriptomic dataset for a non-human primate, the cynomolgus macaque (Nakamura et al., 2016). There are several important observations in this study. Notably, there is a marked difference between pre- and early post-implantation epiblast, as in mouse. Unlike mouse, however, the post-implantation epiblast appears relatively consistent in gene expression for several days extending to early gastrulation. This could indicate that in primates formative-stage cells persist for longer and potentially self-renew. The study also concluded that conventional human PSCs are most closely related to the late post-implantation epiblast in primates, similar to mouse EpiSCs, whereas the candidate human naïve cells (Guo et al., 2016; Takashima et al., 2014; Theunissen et al., 2014) are more similar to the pre-implantation epiblast.

The availability of an *in vivo* reference from cynomolgus and an *in vitro* experimental system in the form of human naïve-like PSCs makes it feasible to address not only whether a formative process occurs, but also whether it is necessary for definitive lineage specification in primates. Significantly, current human naïve cells appear recalcitrant to direct entry into differentiation and are first cultured in primed conditions for several days (Guo et al., 2016; Takashima et al., 2014). It will be important to characterise events during this period and determine the optimal conditions for transitioning naïve cells into full lineage competence. Of note, the formative phase is likely to last longer than in rodent embryos, which advance more rapidly to gastrulation (Fig. 2). This extended time window could facilitate analysis and characterisation. Comparative studies in other mammals, such as pig and rabbit, which progress to gastrulation before implantation, will also be instructive.

Interestingly, a hierarchical population structure has been reported for conventional human PSC cultures (Hough et al., 2014). It will be informative to determine whether the primitive cells at the apex of the hierarchy display features of formative pluripotency. It will also be instructive to examine whether the frequency of such cells changes in various culture conditions that have been reported to alter pluripotent stem cell properties. Identifying cell surface markers and/or knock-in fluorescent reporters of the formative phase will be invaluable for such analyses.

A major challenge will be to derive and propagate stem cells representative of formative pluripotency. This will depend on whether a stable attractor state (Enver et al., 2009) exists within the formative phase that can then be captured *in vitro*. This is by no means a given. Notably, EpiLCs, which are close to, or may include, formative cells, are considered as a transient population (Hayashi et al., 2011). On the other hand, success in pausing progression at the beginning (naïve ESCs) and end (primed EpiSCs) phases of pluripotency provides a strong precedent. Moreover, the persistence of the post-implantation epiblast in the primate embryo (Nakamura et al., 2016) might endow a greater propensity for *ex vivo* stability and stem cell derivation.

Formative PSCs, if they can be derived, can be expected to have exited the naïve phase but remain lineage neutral. They should have a discrete transcriptional and epigenetic identity embodying the capture of the corresponding formative pluripotency attractor state. Formative PSCs could have advantageous features compared with both naïve and primed stem cell cultures. In human, stability of the embryonic disc epiblast might be reflected in seamless and efficient stem cell derivation under the right conditions. The resultant human formative PSCs might also be genetically and phenotypically more robust than naïve cells, which appear intrinsically less stable in primates than in rodents (Takashima et al., 2014). At the same time, formative PSCs are expected to be more homogeneous than primed cells. They should be directly responsive to inductive cues, in contrast to naïve cells, but potentially with greater consistency and efficiency than primed cells. Finally, current human naïve-like cells are compromised by the erosion of imprints due to demethylation (Pastor et al., 2016). Formative phase cells are expected to have upregulated *de novo* and maintenance methyltransferase activities and should therefore be less susceptible to loss of imprints. A brief period in naïve pluripotency conditions could be sufficient for major epigenome remodelling whilst preserving imprints. Reprogramming somatic cells to naïve status then rapidly converting to formative PSCs could therefore be an attractive option for obtaining robust and unbiased cultures with imprints maintained. A complementary approach would be to establish formative PSCs directly from early human ICM explant cultures, which form a post-implantation epiblast-like structure (Deglincerti et al., 2016; O'Leary et al., 2012; Shahbazi et al., 2016).

## Conclusions

The testable hypothesis of an essential formative phase focusses attention on the mechanisms that confer multi-lineage competence. Naïve and formative pluripotency are globally distinct; the transition between them involves abrupt network dissolution and replacement. By contrast, the progression from formative to primed pluripotency may entail an incremental set of changes and the boundary between these phases might be less distinct. In the embryo, cells transit through pluripotency with high efficiency and fidelity en route to lineage commitment. In the culture environment, however, this sequence could become derailed, contributing to inconsistent, heterogeneous and abortive *in vitro* differentiation. Elucidation of the developmental programme of transitions within the pluripotent compartment is fundamental to understanding how lineage decisions are enabled and executed. In addition, recapitulating this programme *in vitro* might enable improved control and quality of pluripotent stem cell differentiation.
